# Findings of Brain Magnetic Resonance Imaging in Girls with Central Precocious Puberty Compared with Girls with Chronic or Recurrent Headache

**DOI:** 10.3390/jcm10102206

**Published:** 2021-05-19

**Authors:** Shin-Hee Kim, Moon Bae Ahn, Won Kyoung Cho, Kyoung Soon Cho, Min Ho Jung, Byung-Kyu Suh

**Affiliations:** 1 Department of Pediatrics, Incheon St. Mary’s Hospital, College of Medicine, The Catholic University of Korea, Incheon 21431, Korea; kshped@catholic.ac.kr; 2 Department of Pediatrics, College of Medicine, The Catholic University of Korea, Seoul 06591, Korea; mbahn@catholic.ac.kr (M.B.A.); wendy626@catholic.ac.kr (W.K.C.); soon926@hanmail.net (K.S.C.); suhbk@catholic.ac.kr (B.K.S.); 3 Department of Pediatrics, Yeouido St. Mary’s Hospital, College of Medicine, The Catholic University of Korea, Seoul 07345, Korea

**Keywords:** central precocious puberty, girls, headache, brain MRI

## Abstract

In the present study, the results of brain magnetic resonance imaging (MRI) in girls with central precocious puberty (CPP) were compared those in with girls evaluated for headaches. A total of 295 girls with CPP who underwent sellar MRI were enrolled. A total of 205 age-matched girls with chronic or recurrent headaches without neurological abnormality who had brain MRI were included as controls. The positive MRI findings were categorized as incidental non-hypothalamic–pituitary (H–P), incidental H–P, or pathological. Positive MRI findings were observed in 39 girls (13.2%) with CPP; 8 (2.7%) were classified as incidental non-H–P lesions, 30 (10.2%) as incidental H–P lesions, and 1 (0.3%) as a pathological lesion (tuber cinereum hamartoma). The prevalence of positive MRI findings in girls with CPP did not differ from girls with headaches (13.2% vs. 12.2%, *p* = 0.74). The prevalence of incidental H–P lesions in girls with CPP <6 years of age, 6–6.9 years of age, and 7–7.9 years of age was 21.2%, 13.5%, and 9.6%, respectively (*p* = 0.21). Known pathological lesions were detected in only one (3.0%) girl with CPP aged <6 years and in no girls with CPP aged 6–7.9 years. Microadenomas were detected in no girls with CPP aged <6 years and in 5 (1.9%) girls with CPP aged of 6–7.9 years. Our findings call into question the routine use of brain MRI in girls with CPP, especially in girls 6 years or older. Current guidelines recommend a follow-up MRI in cases of microadenoma, but few data exist to support this recommendation for children.

## 1. Introduction

Central precocious puberty (CPP) is defined as the onset of pubertal signs by the activation of the hypothalamic–pituitary–gonadal axis before 8 years of age in girls and before 9 years of age in boys [1]. CPP is categorized as organic when associated with a lesion of the central nervous system (CNS), such as hamartomas and tumors, and as idiopathic when not associated with a CNS lesion [2].

The reported prevalence for unsuspected intracranial lesions in children diagnosed with CPP varies depending on gender, from 8% to 33% in girls [3] and up to 40% in boys [4]. Although there is consensus on performing brain MRIs in all boys and girls younger than 6 years of age with CPP, CNS imaging in girls 6 years of age or older with CPP remains controversial due to the low prevalence of CNS lesions [5]. In addition, the disadvantages of MRI include the high cost, the extended period required for sedation, and the intravenous administration of contrast agents. However, in other studies, the proportion of CNS lesions in girls 6–8 years of age with CPP was reportedly significant and should not be disregarded [6].

Intracranial pathology in patients with a common headache occurs at a rate roughly comparable with the general population [7]. Therefore, to determine the significance of brain imaging in girls with CPP based on the age of onset, the girls who underwent a brain MRI for chronic or recurrent headaches were selected as controls. In the present study, the results of brain MRIs in girls with CPP were compared with girls evaluated for headaches.

## 2. Subjects and Methods

### 2.1. Patients and Settings

**Figure 1:**
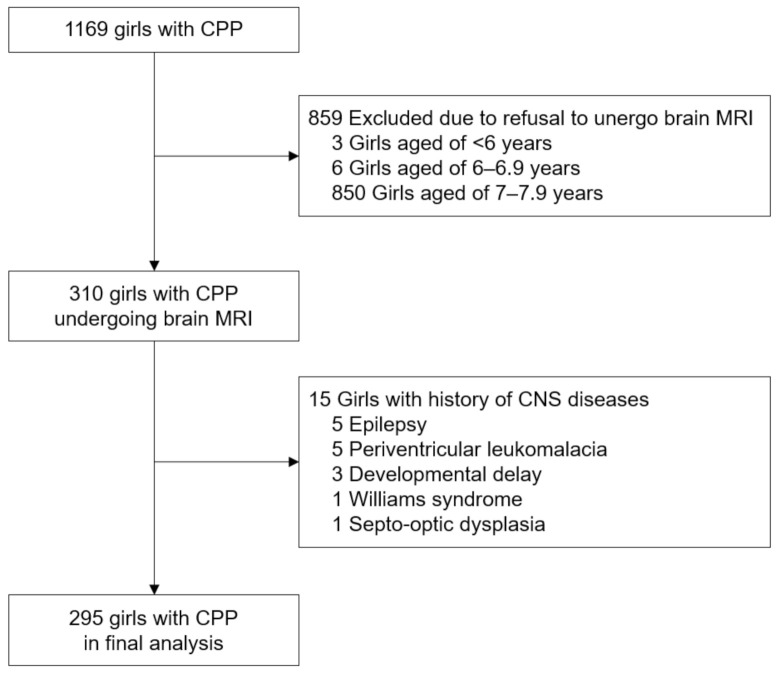
Flowchart of girls with CPP enrolled in the study.

All girls with CPP who visited the pediatric endocrinology clinic at Inchon St. Mary’s Hospital and Yeouido St. Mary’s Hospital from January 2010 to December 2019 were enrolled in the study. Diagnosis of CPP was made based on the following classic criteria: (1) onset of breast development (stage B2 or above according to the Tanner breast stage) before 8 years of chronological age (CA); (2) advanced bone age at least 1 year over CA; and (3) pubertal luteinizing hormone (LH) response >5 IU/L to a gonadotropin-releasing hormone (GnRH) stimulation test [8]. During this period, 1169 girls were diagnosed with CPP and 310 girls underwent sellar MRI. Of these 310 girls with CPP, 15 girls with a history of CNS diseases were excluded. Finally, a total of 295 were included in the analysis. (Figure 1). The CA at pubertal onset in girls diagnosed with CPP was 6.6 ± 0.9 years (range 3.1–7.8 years).

The clinical data of the girls examined for headache, all of whom were referred to the pediatric neurologic clinic of the two hospitals from January 2010 to December 2019, were reviewed. The subjects chiefly reported experiencing chronic or recurrent headache episodes for 1 month or longer, had a normal neurologic examination, and were between 6 and 8 years of age. Patients with known CNS lesions, prior brain surgery, past history of head trauma, seizure, developmental delay, or associated endocrine disorders, previous hormonal therapies, malformations, or other inherited conditions, including congenital adrenal hyperplasia, were excluded. Among the 3,632 brain MRIs analyzed, 205 cases met the criteria. A total of 205 girls with chronic or recurrent headache 6–7 years of age were included in this study. The CA of the girls at evaluation who had chronic or recurrent headache was 7.0 ± 0.6 years (range 6.0–8.0 years). The study was reviewed and approved by the local Institutional Review Board, which has jurisdiction over the local study populations.

### 2.2. Measurements

The initial evaluation of girls with CPP included height, weight, pubertal maturity, bone age (BA), basal plasma estradiol, luteinizing hormone (LH), and follicle-stimulating hormone (FSH) levels before and after GnRH stimulation. At each evaluation, height was measured using a Harpenden’s stadiometer. Weight and height were recorded to the nearest 0.1 kg and 0.1 cm, respectively. Weight and height SDS were calculated using the 2007 Korean National Growth Charts. Body mass index (BMI) was calculated as weight (kilograms)/height (square meters) and was expressed in SDS for CA, according to the 2007 Korean National Growth Charts [9]. Pubertal maturity was assessed according to the Marshall and Tanner criteria [10]. BA was determined according to the atlas of Greulich and Pyle [11].

### 2.3. Brain MRI

Brain MRIs were performed before and after gadolinium-enhanced T1- and T2-weighted images in axial, coronal, and sagittal sections, and a GE scanner with a 3.0 Tesla (MAGNETOM Skyra; Siemens Healthcare, Erlangen, Germany) was used. Thin-section MRI images of the sellar regions (slice thickness of 2.5 mm) were acquired in all patients with CPP, and high-resolution 3D contrast-enhanced MRI images (slice thickness of 1.0 mm) were acquired in all girls with headaches. Dynamic contrast-enhanced imaging of the sellar regions was also acquired in all girls with CPP.

For girls 6 years of age or younger who were diagnosed with CPP, a routine sellar MRI was performed. For girls 7 years of age, the sellar MRI was performed when their parents wanted their child to undergo a sellar MRI or if the girls had signs and symptoms suspected of CNS lesions (Figure 1).

The MRI findings were categorized as follows:

(1) Normal;
(2) Positive:
  (a) incidental: non-hypothalamic–pituitary (non-H–P) or H–P lesions (mild intracranial alterations not involving the H–P region), which are not considered associated with CPP or headache;
  (b) pathological: lesions known to be the cause of or associated with CPP or headache.

### 2.4. Statistical Analysis

The data were analyzed using SPSS version 21.0 (SPSS INC., Chicago, IL, USA), and a *p*-value <0.05 was considered statistically significant. Categorical variables were expressed as frequencies and percentages, and compared using the Chi-square test or Fisher’s exact test, as appropriate. Continuous variables were expressed as means and standard deviations unless otherwise stated.

## 3. Results

### 3.1. MRI Findings of Girls with CPP

The CA at pubertal onset in girls diagnosed with CPP was 6.6 ± 0.9 years (range 3.1–7.8 years). Positive MRI findings were found in 39 girls (13.2%) with CPP; 8 (2.7%) were classified as incidental non-H–P lesions, 30 (10.2%) as incidental H–P lesions, and 1 (0.3%) was classified as a pathological lesion. Among the incidental H–P lesions, there were seven cases of Rathke cleft cysts (RCC), six cases of arachnoid cysts, five cases of microadenoma, four cases of pituitary hypoplasia, three cases of cysts of the pars intermedia, three cases of pineal gland cysts, and two cases of pituitary hyperplasia. The only pathological finding was tuber cinereum hamartoma (Table 1). The patient with tuber cinereum hamartoma visited our clinic due to early breast development (Tanner stage 2) at the age of 4 years and 3 months. She had a significant advanced bone age of 9.5 years, and her peak-stimulated LH levels after GnRH stimulation test were 61 IU/L. No other signs and symptoms associated with hamartoma were noted.

### 3.2. Comparison of Brain MRI Findings between Girls with CPP and Girls with Headache

The CA at evaluation in girls with chronic or recurrent headache was 7.0 ± 0.6 years (range 6.0–8.0 years). The prevalence of positive MRI findings was 25 girls (12.2%) with headache; 12 (5.9%) were classified as incidental non-H–P lesions, 12 (5.9%) as incidental H–P lesion, and 1 (0.5%) was classified as a pathological lesion. Among the incidental H–P lesions, there were seven cases of arachnoid cysts, two cases of microadenoma, two cases of RCC, and one case of pineal gland cyst. The only pathological finding was Moyamoya disease (Table 2).

The prevalence of positive MRI findings in girls with CPP was not different from girls with headache (13.2% vs. 12.2%, *p* = 0.74). There was a trend towards a higher prevalence of incidental H–P lesions in girls with CPP than in girls with headache (10.2% vs. 5.9%, *p* = 0.09).

### 3.3. Brain MRI Findings Based on Age at Pubertal Onset in Girls with CPP

The girls with CPP were categorized into three groups based on age at pubertal onset: <6 years (*n* = 33), 6–6.9 years (*n* = 148), and 7–7.9 years (*n* = 114). The prevalence of positive MRI findings was relatively higher in girls with a younger onset, but without statistical significance. The prevalence of incidental H–P lesions in girls with CPP < 6 years of age, 6–6.9 years of age, and 7–7.9 years of age was 21.2%, 13.5%, and 9.6%, respectively (*p* = 0.21; Table 3). Microadenomas were detected in no girls with CPP aged <6 years and in five (1.9%) girls with CPP aged 6–7.9 years. Known pathological lesions were detected in only one (3.0%) girl with CPP aged <6 years and in no girls with CPP aged of 6–7.9 years. The clinical characteristics (mean age at diagnosis, Tanner state, and BA–CA) and biochemical parameters (basal LH levels, basal FSH levels, peak LH levels, peak LH levels, and peak LH/FSH ratio) did not differ between the patients with positive MRI findings and those without (data not shown).

### 3.4. Follow-Up Brain MRI

Fifteen girls (38.5%) with CPP who had positive MRI findings underwent brain MRI re-evaluation. During the follow-up period (2.2 ± 1.3 years), no lesion showed an increase in size, and signs of neurological or endocrine disorders were not observed in any of the 15 patients. In one patient, the size of microadenoma decreased by 2 mm. Re-evaluation was performed in six girls (50%) with headaches who had incidental H–P lesions, and the size of the pituitary lesions was compared over time; all lesions remained stable.

## 4. Discussion

In the present study, the data of 295 girls with CPP obtained from two centers showed that the incidence of pathological lesions in girls with CPP was lower than in previous reports and that the incidence of positive MRI findings in girls with CPP did not differ from girls with headaches. Known pathological lesions were detected in only one (3.0%) girl with CPP aged <6 years and in no girls with CPP aged of 6–7.9 years.

The findings in the present study showed that a very small portion of girls with CPP (less than 1%) had pathological CNS lesions. Ng et al. reported a high prevalence of neoplasms in CPP girls (15% of 67 subjects) in the United Kingdom between 1990 and 2001 [12]. Cisterino et al. reported brain abnormalities in 18.4% of 304 CPP girls in Italy between 1988 and 1998; however, most patients with brain abnormalities had previously known CNS alterations [3]. According to Mogensen et al., clinical or biochemical parameters were reportedly not sufficiently sensitive and specific to predict brain abnormalities in girls with CPP [6]. In contrast, a very low frequency of pathological findings has been reported in other recent studies [13,14,15]. Incidental findings were observed in 8.2% of 317 CPP girls, and pathological brain lesions were not observed in any patient in Korea between 2003 and 2016 [13]. Chiu et al. reported that pathological findings associated with CPP were detected in only one girl (0.4%) under 6 years of age in Taiwan between 1997 and 2017 [15].

Factors contributing to the timing of puberty include obesity, genetic factors involving familial, ethnic, and sex patterns, and environmental factors, such as nutrition and endocrine-disrupting chemicals [16]. In the Korean National Registry-based epidemiologic study, the annual incidence of CPP in girls increased four-fold from 2008 to 2014 [17]. Similarly, in a Spanish epidemiological study, the annual incidence of CPP in girls increased more than seven-fold from 1997 to 2009 [18]. A greater number of girls may have been diagnosed with CPP over the past decades because pubertal timing has advanced in the general population with no changes in the diagnosis criteria of CPP. In addition, an increase in physician awareness of precocious puberty and the resulting increase in evaluation based on social awareness may be another reason for the high incidence and prevalence of CPP in girls [17]. Therefore, the marked decrease in the incidence of pathological brain lesions could be partly associated with the increased diagnosis of CPP and the earlier onset of normal puberty in girls.

Our data showed the incidence of positive MRI findings in CPP girls was not different from that in headache girls. There was a trend towards more frequent incidental H–P lesions in girls with CPP than in girls with headaches (10.5% vs. 5.9%). This finding should be cautiously interpreted because different MRI protocols were used between the CPP and headache groups. Despite the high resolution of images acquired in both groups (slice thickness of 1.0–2.5 mm), dynamic contrast-enhanced imaging of the sellar regions was acquired only in girls with CPP. The addition of dynamic images to routine MRI images increases the overall detection rate of pituitary microadenoma [19]. Therefore, there is a possibility that some lesions, such as microadenoma, could be missed in patients with headaches.

In girls with CPP, RCC was the most prevalent incidental H–P lesion, followed by arachnoid cyst and pituitary microadenoma. In girls with headaches, arachnoid cyst was the most prevalent incidental H–P lesion, followed by pituitary microadenoma and RCC. Although RCC has been frequently found in patients with CPP, the relationship between RCC and CPP has not been established yet. A previous study evaluated whether incidentally discovered RCC influences the treatment response of CPP [20]. The authors concluded that treatment outcomes for CPP were similar between the CPP patients with and without RCC [20]. In a study of 75 patients diagnosed with RCC who underwent a follow-up MRI, the majority of the lesions were unchanged or decreased in size over time [21].

In this study, microadenomas were detected in 5 (1.9%) of 262 girls with CPP aged 6–7.9 years. The clinical significance of incidental pituitary microadenomas in children is controversial, and there are insufficient data on the long-term outcomes. Pituitary FSH-secreting adenomas were reportedly associated with CPP [22]. In another study, pathological examination of the surgical specimen for pituitary adenoma in girls with CPP showed strong immunostaining for FSHβ [23]. In the 2011 Endocrine Society guidelines, all patients with radiologically-diagnosed pituitary microadenoma were recommended to undergo investigation to evaluate hormone abnormality and to have a follow-up MRI at 1- or 2-year intervals [24]. In studies on adult patients, 3–14% of microadenoma enlarged during the follow-up period [25,26,27]. However, whether adult guidelines provide a suitable framework for follow-up in the pediatric population is unknown. In an MRI study with 44 children based on repeated imaging, significant increases in microadenoma size and hormonal changes were not observed on follow-ups of 4.5 ± 2.6 years [28].

Arachnoid cysts are incidental findings with a prevalence of 1.7–2.6% in children and in most cases are asymptomatic and found incidentally [29,30]. Pineal cysts also frequently occur, from 1.5–4.3% on brain MRIs, and are becoming more common in children and adolescents [31]. Pituitary gland hyperplasia, which is a frequent cause of incidental findings, physiologically occurs during puberty and pregnancy, and frequent hormonal and neuroradiological follow-up appears unnecessary without identifiable abnormalities [32].

In the present study, known pathological lesions were observed in only one (0.3%) girl under 6 years of age. Age <6 years at puberty onset has reportedly been an independent risk factor for pathological CNS lesions in girls with CPP [14,33]. Recently, a meta-analysis including three studies and 687 girls with CPP demonstrated that the pooled prevalence of pathological brain lesions was 25.4% in girls <6 years old and 2.5% in girls 6–8 years old [34]. Conversely, two recent studies reported that the overall prevalence of pathological lesions was low across all age groups (0% [13] and 0.4% [15]), which is consistent with our data (0.3%). Based on these findings, we doubt the need for routine brain MRI in CPP girls aged 6 years or older who do not have signs or symptoms suggestive of intracranial pathology. The low prevalence of known pathological lesions in our CPP girls <6 years should be confirmed by further large-scale studies because of the relatively small number of cases in this age group.

The present study had several limitations. First, the retrospective nature of the study does not allow firm causal conclusions to be drawn. Second, the prevalence of brain lesions in girls with CPP who have not undergone brain MRI is unknown, and long-term follow-up of these patients is required.

In conclusion, the prevalence of positive findings on brain MRI does not differ between girls with CPP and girls with headaches. Known pathological lesions were rarely detected in CPP girls under 6 years of age and not detected in CPP girls aged 6–7.9 years. Microadenomas were detected in no girls with CPP aged <6 years and in five (1.9%) girls with CPP aged of 6–7.9 years. Our data question the routine use of brain MRIs in girls with CPP, especially in girls 6 years or older. Although current guidelines recommend a follow-up MRI in cases of microadenoma, no large studies in children have documented growth of such lesions during follow-up.

## Figures and Tables

**Table 1:** Details of positive brain MRI findings of girls with CPP.

MRI Findings	All Patients (*n* = 295)
**Incidental non-hypothalamic-pituitary legion**	
Neuroglial cyst	1 (0.3)
Callosal lipoma	1 (0.3)
Tornwaldt’s cyst	1 (1.3)
Non-neoplastic cyst	3 (1.0)
Nodule of thalamus	1 (0.3)
Subependymal gray matter	1 (0.3)
**Incidental hypothalamic-pituitary legion**	
Rathke cleft cyst	7 (2.4)
Arachnoid cyst	6 (2.0)
Microadenoma	5 (1.7)
Pituitary hypoplasia	4 (1.4)
Cyst of pituitary pars intermedia	3 (1.0)
Pineal gland cyst	3 (1.0)
Pituitary hyperplasia	2 (0.7)
**Pathological lesion**	
Hypothalamic tuber cinereum hamartoma	1 (0.3)

Data are the number (%) of patients unless otherwise indicated.

**Table 2:** Details of positive brain MRI findings of girls with chronic or recurrent headache.

MRI Findings	All Patients (*n* = 205)
**Incidental non-hypothalamic-pituitary legion**	
Neuroepithelial cyst at the temporal region	1 (0.5)
Empty sella configuration	1 (0.5)
Non-neoplastic cyst	1 (0.5)
Partial agenesis of the corpus callosum	1 (0.5)
Ventricular dilatation	2 (1.0)
Enlarged perivascular space	1 (0.5)
Vascular anomalies	1 (0.5)
Developmental venous anomaly	4 (2.0)
**Incidental hypothalamic-pituitary lesion**	
Arachnoid cyst	7 (3.4)
Microadenoma	2 (1.0)
Rathke cleft cyst	2 (1.0)
Pineal gland cyst	1 (0.5)
**Pathological lesion**	
Moyamoya disease	1 (0.5)

Data are number (%) of patients unless otherwise indicated.

**Table 3:** Brain MRI findings based on age at pubertal onset in girls with CPP.

MRI Findings	<6 years (*n* = 33)	6–6.9 years (*n* = 148)	7–7.9 years (*n* = 114)	*p*-Value
Normal (*n* = 256)	25 (75.8%)	128 (86.5%)	103 (90.4%)	NA
Positive findings (*n* = 39)	8 (24.2%)	20 (13.5%)	11 (9.6%)	0.09
Incidental findings (*n* = 38)	7 (21.2%)	20 (13.5%)	11 (9.6%)	0.21
Non-H–P region (*n* = 7)	1 (3.0%)	4 (2.7%)	3 (2.6%)	>0.99
H–P region (*n* = 31)	6 (18.2%)	16 (10.8%)	8 (7.0%)	0.16
Pathological findings (*n* = 1)	1 (3.0%)	0	0	NA

Data are number (%) of patients unless otherwise indicated. yrs, years; H–P, hypothalamic–pituitary; NA, not applicable.

## Data Availability

The data presented in this study are available on request from the corresponding author. The data are not publicity available due to privacy or ethical restriction.
